# The Association Between Outdoor Temperature During the Past Weeks and Current Fluid Homeostasis

**DOI:** 10.1097/EDE.0000000000001968

**Published:** 2026-03-02

**Authors:** Sofia Enhörning, Olle Melander, Sölve Elmståhl, Gunnar Engström, Mats Pihlsgård, Simon Timpka

**Affiliations:** From the a Department of Clinical Sciences in Malmö, Perinatal and Cardiovascular Epidemiology, Lund University, Malmö, Sweden; b Department of Internal Medicine, Skåne University Hospital, Malmö, Sweden; c Department of Clinical Sciences in Malmö, Lund University, Malmö, Sweden; d Division of Geriatric Medicine, Department of Clinical Sciences in Malmö, Lund University, Malmö, Sweden; e Department of Obstetrics and Gynecology, Skåne University Hospital, Malmö, Sweden.

**Keywords:** Cold environment, Copeptin, Heat, Hydration, Temperature-related mortality, Urine osmolality, Vasopressin, Water intake

## Abstract

**Background:**

It remains unclear why cool temperatures cause more persistent adverse health effects compared with hot weather. Fluid homeostasis may constitute a causal link between past temperatures and adverse health effects. In this study, we investigated the association between past outdoor temperatures and current fluid homeostasis.

**Methods:**

We studied participants from five cohorts during three decades in Sweden (total n = 29,755, age 18–86 years, 50.4% women). We quantified fluid homeostasis through indicators of hormonal regulation (vasopressin biomarker plasma copeptin), urine concentration (urine osmolality), and replenishment of fluid loss (total water intake) and related these parameters to past outdoor temperatures (21 days) using distributed nonlinear lag models.

**Results:**

Past temperatures were nonlinearly associated with current copeptin and urine osmolality. Cool temperatures during days and weeks prior contributed to distinct patterns of high copeptin with concomitant influence on urine osmolality. Overall, a scenario of temperatures of 0 °C for 21 days showed 14.9% (95% confidence interval = 11.5%, 18.3%) higher copeptin levels compared with reference temperatures of 14.3 °C for 21 days. Associations between copeptin and warm temperatures were less complex and of shorter duration, linking elevated temperatures within 24 hours with higher copeptin compared with the reference temperature.

**Conclusions:**

Alterations in human fluid homeostasis may partly explain the observed link between moderately cool outdoor temperatures and adverse health effects weeks later. If so, avoiding altered water balance through moderately increased water intake might mitigate the adverse health effects of cool weather.

Ongoing climate change has made the link between suboptimal outdoor temperature and human health a pressing issue.^1^ The detrimental health effects of extreme temperatures are mediated through both changes in body core temperature and acute physiological adaptations. However, cooler temperatures are linked to a substantially larger proportion of deaths than high temperatures, partly because they are much more frequent.^2^ Still, the pathophysiological mechanisms of this increased risk are not fully known.^3^

Temperature-related changes in fluid homeostasis may constitute a link between past temperatures and adverse health effects. In a recent review of health effects from decreased fluid intake, we identified the hydration marker vasopressin as a possible causal link between decreased hydration and cardiometabolic disease development.^4^ The hormone vasopressin is mostly known as a regulator of fluid homeostasis due to its ability to mediate water reabsorption in the kidney, but it is also involved in several other physiological processes, and elevated levels of the vasopressin surrogate marker copeptin is associated with cardiometabolic disease development and premature mortality.^4^ In a recent study, we found both moderately cold and warm short-term temperatures to be associated with higher concentrations of plasma copeptin.^5^ Another study showed similar nonlinear associations between outdoor temperature and other measures of decreased hydration.^6^ Furthermore, numerous previous studies have reported a nonlinear relationship between outdoor temperature and mortality,^2,7^ with the lowest mortality rates observed around the 75th temperature percentile.^7^ Because the shape of the association between outdoor temperature and mortality is similar to that of the association between outdoor temperature and measures of hydration, we hypothesize that decreased hydration may constitute a mechanistic link between temperature and increased rates of morbidity and mortality.

The vast majority of evidence in the field of temperature-related health effects comes from studies that investigate either effects of extreme temperatures, short-term temperature effects, a limited time period, and/or outcomes that only allow for linkage to the occurrence of major disease events.^7,8^ Here, we aimed to investigate negative fluid balance as a possible mechanism underlying temperature-related disease risk. Our primary aim was to characterize the short- and medium-term relationships between suboptimal temperatures and alterations in human fluid homeostasis using data on complementary indicators of fluid homeostasis (the vasopressin biomarker copeptin, urine osmolality, and water intake) from a large number of participants in distributed lag nonlinear models. This methodology allowed us to disentangle short and medium-term associations (days and weeks) between past temperatures and different measures of hydration. Our findings build upon our previous work, in which we showed a short-term effect of outdoor temperature on the hydration marker copeptin^5^ and address a substantial knowledge gap: whether and how outdoor temperature influences human water balance.

## METHOD

### Participants

The five cohorts used in this study were all based in the city of Malmö, Sweden, between the years 1992 and 2022 (total n = 29,755). Population descriptions of the five cohorts (the Malmö Diet and Cancer – Cardiovascular Cohort, the Malmö Preventive Project, the EpiHealth Malmö Cohort, the Swedish CArdioPulmonary BioImage Study Malmö Cohort, and the Malmö Offspring Study) are provided in Table. All cohorts and the pooling of data from the cohorts have been described in detail previously.^5,9^ In each cohort, copeptin was measured in fasting plasma samples, whereas data on urine osmolality and detailed water intake data were only available in the Malmö Offspring Study. Participants were recruited all year round, but each cohort had a lower inclusion rate in the middle of the summer (due to the Swedish summer holiday). The monthly distribution of participant-inclusions per cohort has been specified previously for all included cohorts,^5^ but updated numbers are presented for the Malmö Offspring Study due to additional recruitment of participants and data analyses since the previous publication (eTable 1; https://links.lww.com/EDE/C317). For all cohorts, written informed consent was obtained from each study participant, and all studies were approved by the local ethics committee. This merged project was approved by the Swedish Ethical Review Authority (Dnr 2020-04422 and 2021-06180-02).

**TABLE.** Sample Description Per Cohort

Characteristics of each of the included study cohorts	
Malmö Diet and Cancer Cardiovascular cohort Baseline data collected in 1992–1994	
N	5,211
Age, years	57.6 (5.9)
Men, n (%)	2,129 (40.9)
Body mass index,^a^ kg/m^2^	25.3 (23.0; 27.9)
Plasma copeptin,^a^ pmol/l	5.2 (3.2; 8.2)
Malmö Preventive Project cohort Baseline data collected in 2002–2006	
N	5,396
Age, years	69.4 (6.2)
Men, n (%)	3,768 (69.8)
Body mass index,^a^ kg/m^2^	26.7 (24.3; 29.4)
Plasma copeptin,^a^ pmol/l	7.2 (4.3; 11.9)
EpiHealth cohort Baseline data collected in 2012–2017	
N	8,217
Age, years	60.8 (8.4)
Men, n (%)	3,656 (44.5)
Body mass index,^a^ kg/m^2^	25.8 (23.5; 28.7)
Plasma copeptin,^a^ pmol/l	5.0 (3.6; 7.8)
Malmö Offspring Study cohort Baseline data collected in 2013–2022	
N	5,245
Age, years	42.1 (14.7)
Men, n (%)	2,523 (48.1)
Body mass index,^a^ kg/m^2^	25.4 (22.8; 28.7)
Plasma copeptin,^a^ pmol/l^b^	5.9 (3.9; 9.1)
Urine osmolality, mosm/kg H_2_O^c^	671 (262)
Water intake, gram/day^d^	2,218 (822)
Swedish CArdioPulmonary BioImage Study cohort (Malmö) Baseline data collected in 2014–2018	
N	5,686
Age, years	57.5 (4.3)
Men, n (%)	2,679 (47.1)
Body mass index,^a^ kg/m^2^	26.6 (24.1; 29.7)
Plasma copeptin,^a^ pmol/l	5.2 (3.6; 8.0)

Values are given as mean (standard deviation) unless otherwise specified.

a Median (25th percentile; 75th percentile).

b N = 4,723.

c N = 4,917.

d N = 3,769.

### Meteorological Data Collection

The city of Malmö, Sweden, is situated by the sea in southern Scandinavia and has a surface area of about 75 km2 without any major elevation. The climate is temperate with mean temperatures around 20 °C in July and August and around 0 °C in January and February (eTable 1; https://links.lww.com/EDE/C317). Consequently, the medium-term (up to a few weeks) temperature variability in Malmö is moderate and without considerable variability across seasons. Air humidity levels span between 70% in the summer and 85% in the winter. In this study, we predominantly used data from one single meteorological station in Malmö (“Malmö A”). We addressed missing temperature-related data as described in the eAppendix 1; https://links.lww.com/EDE/C317. Weather data, including hourly mean temperatures and dew point temperatures during the study period (1992–2022), were obtained from the Swedish Meteorological and Hydrological Institute webpage (http://opendata-download-metobs.smhi.se).

As the physiological response to outdoor temperature is dependent on humidity, we used temperature and dew point temperature to calculate and use apparent temperature as our main exposure, as described in the eAppendix 1; https://links.lww.com/EDE/C317. We calculated the mean apparent temperature during each 24-hour period (from 9 am to 9 am) of the 21 days before sampling or the start of dietary registration. A detailed description of the meteorological data collection and calculation of apparent temperature is found in the eAppendix 1; https://links.lww.com/EDE/C317.

### Measurement of Fluid Homeostasis Parameters

Plasma copeptin was measured once in each participant in fasting plasma samples drawn at the baseline investigation. The blood samples were stored at −80 °C until analysis. Brahms CT-proAVP LIA assay was used in the Malmö Preventive Project and Malmö Diet and Cancer – Cardiovascular Cohort and Brahms Copeptin proAVP KRYPTOR assay was used for analyses in the EpiHealth, Malmö Offspring Study and the Swedish CArdioPulmonary BioImage Study Malmö Cohorts. Plasma was collected in an overnight-fasted state, except in the EpiHealth cohort, where only 6 hours of fasting were required.

In the Malmö Offspring Study cohort, urine osmolality was measured (n = 4,917) and dietary water intake assessed (n = 3,769) in addition to plasma copeptin measurements. Urine osmolality in morning urine samples was measured using a freezing point osmometer. The urine collections followed a standardized procedure, including a comprehensive video instruction provided to participants. The day before a clinic visit, participants were instructed to empty the bladder before going to bed, then collect any urine during the night together with the first morning urine. The urine samples were stored at −80 ºC until analysis. Dietary water intake was assessed by a diet record called “Riksmaten 2010,” a validated 4-day web-based record tool developed by the Swedish National Food Administration.^10^ Total water intake was calculated based on the reported intake of both water from beverages and food moisture. The day when diet registration started was the date used when analyzing the effect of temperature on water intake.

### Statistics

The analytic data set included all participants who had complete data on sampling date, temperature data, age, sex, indicator of fluid homeostasis, and body mass index. Copeptin was log-transformed due to a skewed distribution, and *z*-scores were calculated, standardized for sex and cohort. To assess the influence of temperature 21 days before biomarker collection on fluid homeostasis, we applied distributed lag nonlinear models as previously described.^11^ To facilitate interpretation, we henceforth refer to “complete days before sampling” instead of lag. The modeling approach allowed us to estimate the contribution of the mean apparent temperature during each of the 21 days preceding the sampling of each fluid homeostasis indicator. Unless otherwise indicated, all temperature – fluid homeostasis comparisons are made with 14.3 °C as reference. For a detailed description of methods, please see eAppendix 1; https://links.lww.com/EDE/C317. All models were adjusted for age, sex, body mass index, day of the week of outcome registration, and cohort.

### Additional Analyses

As the health effects of temperature are likely to differ by age and sex, we repeated the main analyses stratified by age (≤60 years and >60 years) and by sex, respectively. As urine osmolality and water intake were measured in the Malmö Offspring Study cohort, we separately assessed the influence of temperature on plasma copeptin in the Malmö Offspring Study cohort. We also repeated our main analyses with absolute outdoor temperature instead of apparent temperature as the main exposure. Finally, we performed sensitivity analyses in which we controlled for season (sine and cosine terms) and long-term time trends (10 degrees of freedom for the entire period). SAS 9.4 was used for all statistical analyses.

## RESULTS

The mean age of the pooled sample (n = 29,755) was 57.9 years, and 50.4% of the study participants were women (Table).

### The Association Between Cool Temperature and Fluid Homeostasis

**FIGURE 1.**
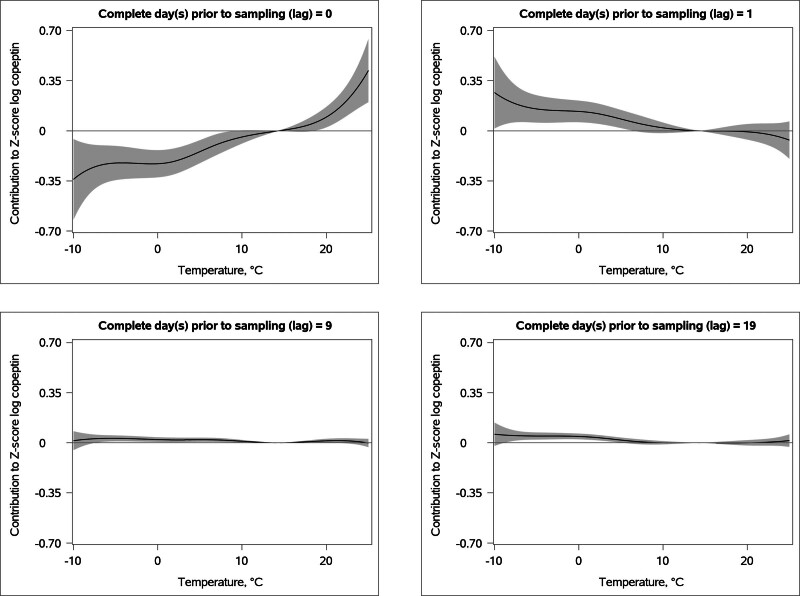
Effect of temperature on plasma copeptin (measured in all cohorts) at different numbers of complete days prior blood sampling. All presented relative to the reference temperature at 14.3 °C. The grey zones denote the 95% confidence intervals.

**FIGURE 2.**
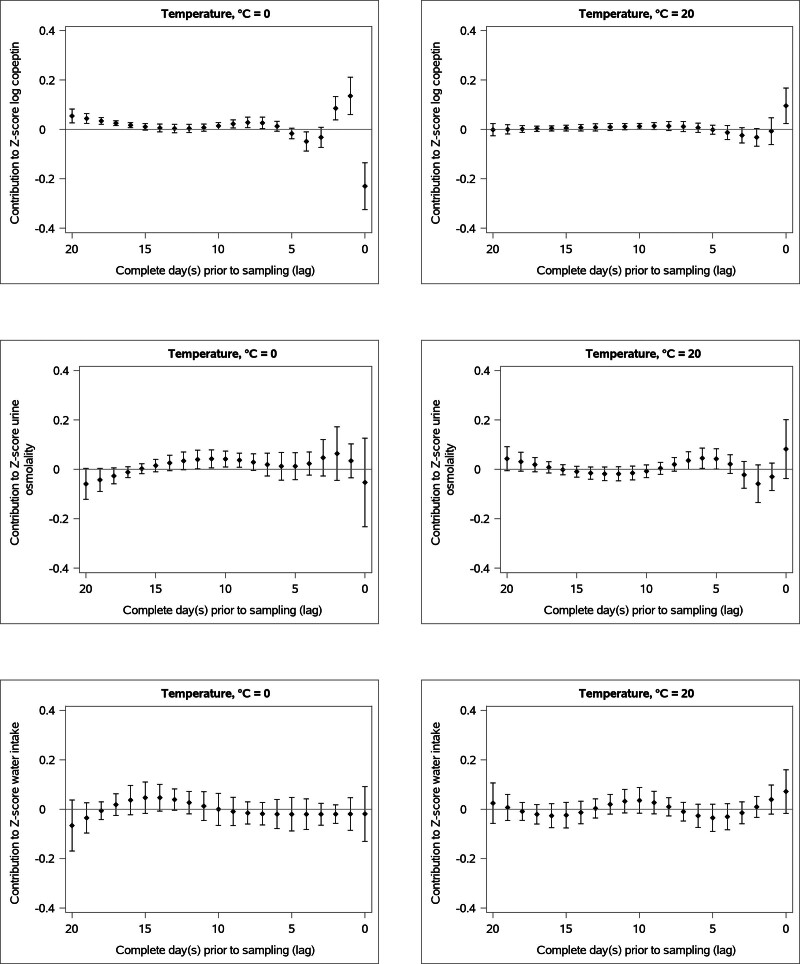
Effect of temperatures of 0 °C and 20 °C on each measured hydration marker (plasma copeptin, urine osmolality, and water intake) at different numbers of complete days prior sampling. All presented relative to the reference temperature at 14.3 °C. The bars denote the 95% confidence intervals. Plasma copeptin measured in all cohorts, water intake and urine osmolality measured in the Malmö Offspring Study cohort only.

The associations between cool temperatures and fluid homeostasis were complex and had a long duration. Cooler temperatures were associated with a short-term and transient plasma copeptin decrease and with increased measures of fluid homeostasis (plasma copeptin and urine osmolality) after a slight delay (Figures 1 and 2, eFigures 2 and 3; https://links.lww.com/EDE/C317). Freezing temperature (0 °C) in the most recent 24 hours before sampling was associated with 14.5% (confidence interval [CI] = 19.8, 8.76) decrease in plasma copeptin compared with the reference temperature (14.3 °C). However, the same temperature (0 °C) one complete day (i.e., 24–48 hours) before sampling was instead associated with 9.65% (CI = 4.17, 15.4) higher plasma copeptin compared with the reference temperature (calculated from data graphically shown in Figure 1). We observed two additional periods of cold-associated plasma copeptin elevation, 7–10 and 16–20 complete days before sampling (Figures 1 and 2). Overall, a scenario of 0 °C temperatures for 21 days was associated with 14.9% higher copeptin levels compared with reference temperatures of 14.3 °C for 21 days (calculated from data graphically shown in eFigure 1; https://links.lww.com/EDE/C317).

The link between cooler temperatures and higher urine osmolality was delayed (eFigures 4 and 5; https://links.lww.com/EDE/C317). For example, a temperature of 0 °C 7–12 complete days before sampling was associated with 0.21 SD (CI = 0.05, 0.36) higher urine osmolality compared with the reference temperature (calculated from data graphically shown in Figure 2). Furthermore, there was a tendency towards a cold-associated decrease in urine osmolality, which was considerably delayed; a temperature of 0 °C 16 to 20 complete days before sampling was associated with 0.13 SD (CI = −0.03, 0.30) lower urine osmolality compared with the reference temperature (calculated from data graphically shown in Figure 2). The cumulative effect of temperature 21 days before sampling on urine osmolality is shown in eFigure 1; https://links.lww.com/EDE/C317, with increased urine osmolality at moderately cold temperatures (0 °C) and decreased urine osmolality at even colder temperatures relative to the reference temperature of 14.3 °C.

We did not find an association between cooler apparent temperatures and dietary water intake (Figure 2, eFigures 6 and 7; https://links.lww.com/EDE/C317). The cumulative effect of temperature 21 days before sampling on water intake is shown in eFigure 1; https://links.lww.com/EDE/C317.

### The Association Between Warm Temperature and Fluid Homeostasis

The link between warm temperatures and fluid homeostasis had a rapid onset and shorter duration. A warm temperature in the most recent 24 hours (0 days) before sampling was associated with a transient increase in plasma copeptin (Figures 1 and 2, eFigures 2 and 3; https://links.lww.com/EDE/C317) and a tendency towards elevated urine osmolality and water intake (Figure 2). More specifically, plasma copeptin concentration was 6.72% (CI = 1.65, 12.0) higher with a temperature of 20 °C, the most recent 24 hours before sampling, compared with the reference temperature, 14.3 °C (calculated from data graphically shown in Figure 2). Urine osmolality was also elevated if the temperature was 20 °C approximately a week before sampling (Figure 2). The cumulative effect of a temperature of 20 °C over 21 days was a 8.82% (CI = 1.19, 17.0) higher plasma copeptin concentration compared with 21 days with the reference temperature (calculated from data graphically shown in eFigure 1; https://links.lww.com/EDE/C317), whereas we did not observe an association between cumulative heat and urine osmolality (eFigure 1; https://links.lww.com/EDE/C317).

We also did not observe an association between warmer apparent temperature and dietary water intake (Figure 2, eFigures 6 and 7; https://links.lww.com/EDE/C317). However, as described below in the additional analyses, we observed an increase in water intake following warm temperatures when crude instead of apparent temperature was used as the exposure. The cumulative effect of apparent temperature 21 days before sampling on water intake is shown in eFigure 1; https://links.lww.com/EDE/C317.

### Additional Analyses

When the influence of temperature on plasma copeptin was separately analyzed in the Malmö Offspring Study cohort, the results strongly resembled the main analyses (eFigures 8–10; https://links.lww.com/EDE/C317).

When the main analyses were repeated but stratified by age (≤60 years and >60 years) and sex, respectively, the cumulative effects of temperature on plasma copeptin and urine osmolality were similar to the nonstratified estimates in each stratum (eFigures 11 and 12; https://links.lww.com/EDE/C317). However, both the cumulative heat and cold effects on elevated plasma copeptin were more pronounced in women than in men (eFigure 11; https://links.lww.com/EDE/C317), and older individuals (>60 years) had a significantly lower water intake as a result of cold temperatures as when compared with younger individuals (≤60 years) (eFigure 12; https://links.lww.com/EDE/C317). When using absolute temperature instead of apparent temperature in our main analyses, we found no major difference in results (eFigures 13 and 14; https://links.lww.com/EDE/C317), but we observed a short-term (24 hours) increase in water intake following warm temperatures (20 °C) compared with the reference temperature (eFigure 14; https://links.lww.com/EDE/C317). When season (sine and cosine terms) was introduced into the model investigating the cumulative effect of temperature the past 3 weeks on different indicators of fluid homeostasis, the associations remained largely similar, although the cold effect was attenuated (eFigure 15; https://links.lww.com/EDE/C317). Introducing long-term trends into the model, we also found that the effect remained similar, although marginally attenuated (eFigure 16; https://links.lww.com/EDE/C317).

### Summary of Main Findings

**FIGURE 3.**
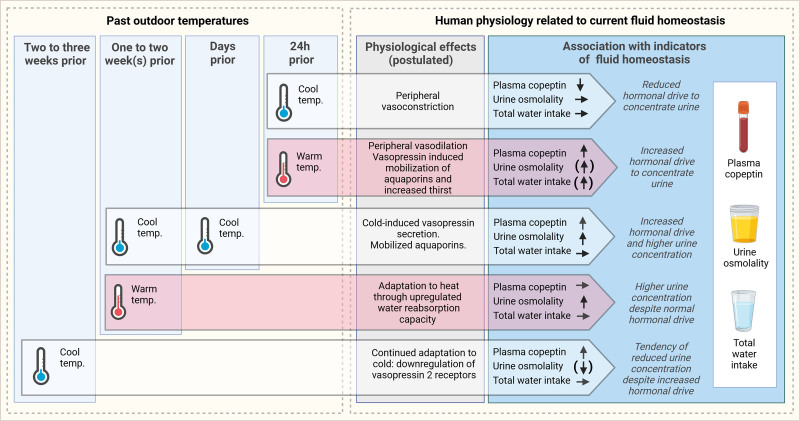
Summary of the effects of past outdoor temperatures on indicators of fluid homeostasis (plasma copeptin, urine osmolality, and water intake) according to our data. This figure was created with BioRender.com

Based on the results above, we identified four distinct periods before sampling (24 hours prior, 1–2 complete days, 1–2 weeks, and 2–3 weeks) with a substantial impact of outdoor temperature on either plasma copeptin or urine osmolality (Figure 3).

## DISCUSSION

A key finding of this study is that cool temperatures were associated with suboptimal and medium-term alterations of human fluid homeostasis over multiple weeks. In contrast, warm temperatures were associated with an altered fluid homeostasis with a more acute onset and of shorter duration. Both cool and warm temperatures have previously been associated with several different hydration markers.^6^ A similar *J*- or *U*-shaped pattern has also been observed in previous literature investigating the effect of temperature on mortality rates. Our findings are consonant with other studies on outdoor temperature during the weeks before incident death in several regions and countries with a wide distribution of climate patterns (tropical, subtropical, and temperate), which suggest that the effects of cool temperature on mortality appear after a delay and last for several days to weeks, while heat-related mortality appears immediately.^7,12^

Our current study substantially deepens and expands on our previous findings of a short-term (within 24 hours) link between temperature and copeptin.^5^ Here, we now use data from the Malmö Offspring Study cohort on two additional indicators of fluid balance to investigate how the outdoor temperature during days and weeks before sampling contributes to distinct patterns of fluid homeostasis, and we also apply more sophisticated analytic models allowing for analysis of specific lags during weeks before the biomarker collection. Our current data suggest rapid and transient physiological responses following warm weather and more sustained physiological processes following cool weather.

Vasopressin is mostly known as a regulator of fluid homeostasis by stimulation of vasopressin 2 receptors in the kidney, thereby mediating water reabsorption in the renal collecting ducts.^13^ Vasopressin is also involved in several other processes mediated through the two other vasopressin receptors (V1a and V1b), affecting metabolic and cardiovascular traits, including platelet aggregation,^14^ vasoconstriction,^15^ glucagon secretion,^16^ adrenocorticotropic hormone release,^17^ and cortisol secretion.^18^ Furthermore, some epidemiological studies have suggested copeptin as a useful marker of premature mortality,^19,20^ and previous experimental and genetic studies suggest a causal link between elevated vasopressin and metabolic disease development.^21–23^ We thus hypothesized that vasopressin, through its diverse hormonal effects, may constitute a mechanistic link between temperature and increased rates of morbidity and mortality, which can span across weeks.

Another possibility is that elevated levels of plasma copeptin in this study simply mirror a negative fluid homeostasis, and that physiological responses to a negative fluid homeostasis other than vasopressin secretion are the link between outdoor temperature and disease risk. For example, it is suggested that direct effects of elevated p-sodium or accumulation of organic osmolytes may play a role in disease risk related to underhydration.^4^

Our results support that cool and warm temperatures affect human fluid homeostasis differently and imply that several underlying mechanisms are involved in the temperature-related physiological responses. In the following section, we outline underlying mechanisms that might explain the temporal patterns and levels of hydration markers that we observe in response to cool and warm temperatures.

The peak in plasma copeptin occurring within 24 hours after exposure to warm temperatures might be linked to a baroreceptor-mediated vasopressin secretion due to peripheral vasodilation leading to decreased effective blood volume,^24^ even though a component of osmotically induced vasopressin secretion is also possible (due to, e.g., sweat-related loss of water). Analogously, the cold-related initial but rapidly transient reduction in plasma copeptin might be related to decreased baroreceptor-mediated vasopressin secretion due to peripheral vasoconstriction, resulting in increased effective blood volume. A small experimental study in humans found vasopressin to decrease after acute cold exposure,^25^ and suggested rapid changes in vasopressin to be baroreceptor-mediated and thus due to redistribution of blood volume.

The rapid transition of the heat-related vasopressin release might be part of a counterbalancing response mediated by vasopressin itself, as it reduces body temperature and water-loss. This response includes increased thirst, decreased urine output, and increased sweating. While a short-term (within 24 hours) increase in water intake and decrease in urine output was, however, not convincingly shown in our main analyses, we observed a short-term increase in water intake following warm outdoor temperatures (20 °C) in additional analyses.

Based on our findings, we speculate that the more delayed onset of elevated plasma copeptin as a response to cold is due to a negative fluid homeostasis. We found a concomitant increase in plasma copeptin and urine osmolality, two out of very few reliable biomarkers of chronic underhydration,^4^ around 8–13 days after onset of cold temperature. Our water-intake data did not support that water intake decreased as a response to cold temperatures, but recent evidence from a study designed to investigate seasonal variation in water intake found a significantly lower water intake during the cold season.^26^ The underlying mechanisms are not known, but we have previously speculated in several potential explanations behind cold-related underhydration,^5,9^ for example, better thirst-quenching effects from cold versus warm drinking water,^27^ social drinking patterns, potential thirst-suppressant effects of darkness, and cold diuresis.^8^

We found an increase in urine osmolality as a response to moderate cold exposure around 2 weeks (10–13 days) before urine sampling, but a tendency towards decreased urine osmolality when cold exposure occurred 3 weeks prior urine sampling. The delayed decrease in urine osmolality as a response to cool temperature resembles the adaptive responses observed in rats exposed to chronic cold, and may be related to increased diuresis due to downregulation of vasopressin 2-receptors in the renal medulla.^28^ Thus, long-term adaptations at the receptor level may partly explain cold-related underhydration.

Around 1 week after exposure to warm temperatures, we found an increased urine osmolality, which we hypothesize is a result of more readily mobilized water reabsorption capacity. This may be due to mobilized aquaporins or urea recycling, and our result points to possible medium-term physiological adaptations induced by warm weather to optimize effective blood volume. This is supported by previous literature stating that athletes need more than 1 week of heat acclimatization to achieve almost complete cardiovascular adaptation.^29^ On the other hand, plasma copeptin concentration was only altered acutely, but not days-weeks after heat exposure. In line with this finding, vasopressin was not previously reported to be altered as a result of heat acclimation,^30^ and heat not associated with increased mortality rates 1 week after exposure.^31^

Studies on the relevance of outdoor temperature on mortality have used a variety of temperature measurements. In concordance with our previous work, and as humidity is relevant for the discomfort and physiological response associated with ambient temperature,^32,33^ we used apparent temperature as our main temperature exposure.

To investigate the delayed relationship between temperature and fluid homeostasis indicators, we used an established approach that allows for limited prior assumptions about the shape of the temperature-outcome curve over the lag period. Temperature knots were primarily chosen to harmonize with our previous work in which the 75th temperature percentile was found to reflect the minimum copeptin concentration in a short-term time frame (up to 24 hours lag).^5^ The 75th temperature percentile has previously been shown to approximately indicate the temperature with the lowest associated mortality rate, that is, the minimum mortality temperature.^7^ As previous studies have found that the minimum mortality temperature is closer to the 90th temperature percentile in a temperate climate, we also added a knot at the 90th temperature percentile.^34^

In this study, the finding that the cumulative heat and cold effect on elevated plasma copeptin was more pronounced in women than in men indicates that the temperature interval associated with lower copeptin concentrations is narrower in women.

As medium- and long-term temperature shifts are tightly linked to seasonal shifts, we did not include adjustment for season in our main analysis. Furthermore, habitually adjusting for season in temperature-outcome analyses is controversial.^35^ Thus, the finding that the association of temperature with fluid balance remained fairly similar even though the cold effect was attenuated after adjustment for season might mainly reflect that adjustment for season is a proxy for adjustment for medium-term and long-term temperature shifts. In other words, the attenuated cold-related effect after adjustments for season is expected since medium-term outdoor temperature variation itself is to some extent a measure of seasonal shifts, whereas the remaining effects after seasonal adjustment would represent the changes in fluid balance that can be explained by within-season temperature variability.

Due to the varying dates of enrollment of study participants, which for the copeptin analyses spanned across 30 years, we additionally adjusted for longer term trends than season to create a model that would capture the changes in hydration parameters over time that were not related to season, and found that the association between temperature and measures of fluid balance remained after adjustments for longer term trends.

### Limitations

In this study, we only include participants from one location living in a temperate climate, and the results may thus not be perfectly generalizable to other climate types. There is a known tendency towards a healthy selection bias in at least some of the Malmö cohorts^36,37^; however, most human studies investigating the effect of temperature on fluid balance in humans have an experimental design and thus only include a very small subset of the population. Another limitation of this study was that, although it was collected through structured and standardized reporting, total water intake was self-reported. Adjustment for season as such can be seen as adjustment for a range of variables with seasonal variation other than longer temperature lags, and therefore, we cannot rule out that our attenuated results after seasonal adjustment were partly influenced by other factors with potential seasonal variation, such as physical activity. Finally, copeptin was measured with two different laboratory methods, which differed between cohorts. However, when using standardized *z*-scores, we previously found that the temperature-copeptin-curve was comparable across cohorts.^5^

## CONCLUSIONS

Cool temperatures during days and weeks prior sampling contributed to distinct patterns of fluid homeostasis characterized by higher copeptin and urine osmolality. These findings suggest a link between cool temperatures and delayed adverse health effects. In contrast, warm temperatures exhibited associations with patterns of fluid homeostasis that were less complex and of shorter duration compared with cool temperatures. Alterations in human fluid homeostasis may constitute an actionable link between moderately cool outdoor temperatures and adverse health effects weeks later.

## Supplementary Material


