# Supplementary material for: The Association Between Outdoor Temperature During the Past Weeks and Current Fluid Homeostasis
Source: Epidemiology. 2026 Mar 2;37(4):447–55. doi: 10.1097/EDE.0000000000001968 (PMC13218588; doi:10.1097/EDE.0000000000001968)
Supplement: Supplementary file 1 [file ede-37-447-s001.pdf]

Supplement to “The association between outdoor temperature during the past weeks and current fluid homeostasis” by Enhörning *et al.*

Table of contents

Supplemental Methods 2
Supplemental Tables 8
Supplemental Figures 9
Supplemental References 25

Supplemental Methods

Temperature data

Data collection

We collected data on temperatures and dew point temperatures in Malmö during the period of participants' blood sampling, urine collection and water intake registration in the city (1992–2022). Detailed data on temperature and dew point temperature from a meteorological station in Malmö ("Malmö A") were obtained through freely available download via Swedish Meteorological and Hydrological Institute webpage (`http://opendata-download-metobs.smhi.se`). To calculate mean apparent temperature during the 24 hour period prior to 9 am on a given day, we used data points on temperature and dew point temperature every 3 h from 9 am the day before to 9 am on the day under consideration. To calculate apparent temperature, defined as a person's perceived air temperature, we used the following formula:

$$-2.653 + 0.994 \times \text{air temperature (°C)} + 0.0153 \times \text{dew point temperature (°C)}.$$

Handling of missing temperature registrations

Of a total of 10,958 days under consideration, 104 days (0.95%) lacked at least one temperature or dew point registration. We imputed missing temperature using a previously described method (see Katsouyanni K. *et al.* [1]). In short, temperature and dew point data for the most adjacent meteorological station "Malmö-Sturup Airport" were used to fill in the missing temperature data. Temperature (apparent or ordinary) at "Malmö A" on a certain day was regressed on corresponding temperature at "Malmö-Sturup Airport" on the same day, with adjustment for seasonality. E.g., R-square for the regression model was 0.994 for apparent temperature. When observation was missing for "Malmö A" we used what was predicted by the regression model. In this way we were able to fill in data for 54 days. We thus lacked apparent temperature registration for 50 out of 10,958 days (0.46%). After imputation, we (e.g.) include 29,137 out of 29,233 copeptin measurements (99.7%) in regression analyses. We find these numbers satisfactory and do not proceed with further imputation.

Statistical modelling

Main analysis

Outcomes were checked for skewness. We decided to apply a logarithmic transformation to plasma copeptin, as we judged that its skewness could possibly reduce performance of subsequent regression analyses. Water intake and urine concentrations were left unchanged. Z-scores based on cohort and sex were constructed for all indicators of fluid homeostasis. All regression analyses were based on least-squares estimation and applied an asymptotic heteroscedasticity consistent covariance estimator. The goal of the modelling was to assess the influence on outcome of temperature in the period 0-20 days prior to outcome registration. To this end, distributed lag non-linear models (dlnm) were applied. These models rest on a so-called cross-basis over exposure dimension and temporal distance to outcome registration (often referred to as lag) dimension (Gasparrini A. *et al.* [2]). Cross-bases are produced by choosing transformations (bases) in

exposure and temporal distance dimension and then applying a mathematical recipe to combine (cross) the transformations/bases.

Tentative models were constructed in a semi-experimental fashion. Temperature was considered on a continuous scale. Five different B-spline models are combined with nine different natural cubic spline lag-models (lag ranging between 0 and 20 days) resulting in a total of 45 candidate continuous temperature-lag cross-basis models. In temperature dimension tentative knots were located according to 1 to 5 below.

1. 25th, 50th and 75th temperature percentile
2. 25th, 50th, 75th and 90th temperature percentile
3. 10th, 20th, 30th, 40th, 50th, 60th, 70th, 80th and 90th temperature percentile
4. Four locations equally spaced between minimum and maximum temperature
5. Nine locations equally spaced between minimum and maximum temperature

In temporal distance (lag) dimension, external knots were placed at 0 and 20. We considered using three, four and eight internal knots. These tentative knots were located at (n denotes number of internal knots)

1. $k \times 20/(n + 1)$, k = 1 to n
2. $\exp(k \times \log(20)/(n + 1))$, k = 1 to n
3. $\exp(k \times \log(21)/(n + 1)) - 1$, k = 1 to n

Method 2 places more knots close to zero than Method 1 does. Method 3 does even more so (Method 2 and Method 3 are quite similar, except close to zero). We provide an example based on SAS below showing how to construct the cross-basis corresponding to Method 2 in temperature dimension and Method 2 for n = 3 in temporal distance dimension.

After this initial step, analyses with adjustment for the co-variables age, sex, BMI, day of the week of outcome registration, and cohort were run for all 45 tentative models (for each outcome under consideration).

In line with previous studies, the final models for analysis and presentation was the candidate model corresponding to minimal Akaike information criterion.

Additional analysis for water intake

For water intake, we had access both to an average value per individual (included in the main analysis), and individual reports per day per individual (in total four water intake registrations per individual). To ensure that no important information was lost when averaging the four measurements, these data were analyzed separately. First, Z-scores based on sex were computed. Then we conducted a repeated measurements regression analysis with an unstructured within individual covariance matrix and cross-basis and adjustment as in the final main analysis. This analysis revealed nothing more than what came out of the main analysis.

Interpretation of presentation of dlnm analyses

We present the results of the dlnm analyses through various graphs. The graphs are of two types. Either we present a cumulative (over the entire period of 21 days) effect of temperature relative to the reference temperature at 14.3 degrees Celsius (see e.g., Supplemental Figure 1), or a local effect at a particular temporal distance to outcome sampling (see e.g., Supplemental Figure 2). The former can be interpreted as the difference in expected outcome between two otherwise identical individuals where one experiences the specified temperature and the other the reference temperature through the entire period of 21 days. The latter can be interpreted as the difference between two individuals, who experience the same temperature except at the particular temporal distance, where one is exposed to the specified temperature and the other to the reference temperature. In the results section of the paper, we go from results for Z-score of logarithm of copeptin presented in Figure 1, Figure 2 and Supplemental Figure 1 (top panel) to corresponding estimates, lower confidence limits (LCLs) and upper confidence limits (UCLs) for relative changes (relative to 14.3 °C) in copeptin through the formulas

Estimate of relative change =100 × (exp(0.68 × Estimate in figure) - 1)
LCL of relative change =100 × (exp(0.68 × LCL in figure) - 1)
UCL of relative change =100 × (exp(0.68 × UCL in figure) - 1)

(the overall standard deviation of logarithm of copeptin in our sample is 0.68). Let us show where these formulas come from. Let y_i and y_j be the copeptin measurements for an individual at an arbitrary temperature and at 14.3 degrees Celsius, respectively. The individual is assumed to come from a population with mean of logarithm of copeptin μ and corresponding standard deviation σ. The regression model is

$$(\log y_i - \mu)/\sigma = X_i\beta + \varepsilon_i$$
$$(\log y_j - \mu)/\sigma = X_j\beta + \varepsilon_j,$$

where X_i and X_j are design vectors, β is a vector of regression parameters and ε_i and ε_j are error terms. It is not too hard to see that the equations above are equivalent to

$$y_i = \exp(\sigma(X_i\beta + \varepsilon_i) + \mu)$$
$$y_j = \exp(\sigma(X_j\beta + \varepsilon_j) + \mu),$$

which in turn yields (we let $\mathbb{E}$ denote mathematical expectation operator)

$$\frac{\mathbb{E}y_i - \mathbb{E}y_j}{\mathbb{E}y_j} = \frac{\exp(\sigma X_i\beta)\exp(\mu)\mathbb{E}\exp(\sigma\varepsilon_i)}{\exp(\sigma X_j\beta)\exp(\mu)\mathbb{E}\exp(\sigma\varepsilon_j)} - 1 = \exp(\sigma(X_i - X_j)\beta) - 1,$$

where we in the last step assumed that ε_i and ε_j have the same distribution (not assumed in the statistical estimation of β where heteroscedasticity is allowed). We conclude by noting that in view of $\mathbb{E}(\log y_i - \mu)/\sigma - \mathbb{E}(\log y_j - \mu)/\sigma = (X_i - X_j)\beta$, the last display is equivalent to

$$\frac{\mathbb{E}y_i - \mathbb{E}y_j}{\mathbb{E}y_j} = \exp(\sigma(\mathbb{E}(\log y_i - \mu)/\sigma - \mathbb{E}(\log y_j - \mu)/\sigma)) - 1,$$

establishing the links between estimates and confidence limits shown above. The SAS code below shows how to construct data required to produce plots.

SAS code example

Temperature data should be in a long format and include the variables “date”, “lag” and some variable representing temperature. Lag data should be in a long format and include the variable “lag”. Variable names seem self-explanatory. Transformed data (prior to crossing) should also contain “lag” and “date” (temperature) and “lag” (temporal dimension). As can be seen below, the construction rests on the macro “cross”, that in addition to names and locations of data sets containing transformed data in temperature and temporal distance dimensions, requires the syntax used for naming transformed variables. E.g., below, transformed values of temperature are stored in “x_1”,...,“x_7” and we thus tell SAS to look for variables whose names contain “x_”. Graphical presentations of analyses are based on the macro “cross_score” that creates data used to score models along appropriate grids over temperature and temporal distance. Input temperature data to this macro should contain “lag”, “x” (representing temperature) and transformed variables for each level of “lag” and “x” (note that these do not depend on the value of “lag”). Input lag data should contain “lag” and transformed variables.

```
SAS CODE:
/*Transform in temperature dimension. Temperature is stored in variable x*/
proc transreg data = temperature design noprint;
model bspline(x/knots = 1.52261 6.970986 14.30823 18.22015
exknots = -13.2689 28.58215 degree = 3) ;
output out = xx(drop = x_0);
id date lag;
run;
/*Transform in temporal distance dimension*/
proc glmselect data = lag outdesign(addinputvars fullmodel) = laglag;
effect lag_ = spline(lag/details naturalcubic basis = tpf()
knotmethod = list(0,2.114743,4.472136,9.457416,20));
model lag =  lag_ ;
run;
/*The macro below takes as arguments the names and locations of the data sets
required to form the cross-basis, the naming syntax for relevant variables and what to
name the output data containing the cross basis.
The names of crossed variables in the output are automatically determined
by variable names in the input data*/
%macro cross(inx=,inlag=,inxcat=,inlagcat=,xsyntax=,lagsyntax=,out=);
proc sql noprint;
select distinct name into :listx separated by ' ' from dictionary.columns
where libname = "%upcase(&inxcat)"
and memname = "%upcase(&inx)" and upcase(name) contains "%upcase(&xsyntax)" order by varnum;
select distinct name into :listlag separated by ' ' from dictionary.columns where
libname = "%upcase(&inlagcat)"
and memname = "%upcase(&inlag)" and upcase(name) contains "%upcase(&lagsyntax)" order by varnum;
create table &out as select distinct
%let i = 1;
%do %while (%scan(&listx,&i,' ') ne %str());
%let j = 1;
%do %while (%scan(&listlag,&j,' ') ne %str());
sum(a.%scan(&listx,&i,' ')* b.%scan(&listlag,&j,' ')) as
%scan(&listx,&i,' ')_%scan(&listlag,&j,' ') ,
%let j = %eval(&j+1);
%end;
```

```
%let i = %eval(&i+1);
%end;
a.date
from
&inx as a inner join &inlag as b on a.lag = b.lag
group by a.date;
quit;
%mend;
/*We run the score macro with input "xx" and "laglag", both located in the "work" directory.
We instruct SAS to use variables with names containing "x_" in the temperature dimension
and variables with names containing "lag_" in the temporal distance dimension.
Output is stored in "cross_basis", located in the "work" directory.*/
%cross(inx=xx,inlag=laglag,inxcat=work,inlagcat=work,xsyntax=x_,lagsyntax=lag_,
out=cross_basis);
/*Generate data to score model. Used in construction of graphs*/
/*Generate data containing combinations of suitable temperature values and lags*/
data x;
do m = 0 to 350;
x = -10 + m/10;
do lag = 0 to 20;
output;
end;
end;
drop m;
run;
/*Transform these data in temperature dimension*/
proc transreg data = x design noprint;
model bspline(x/knots = 1.52261 6.970986 14.30823 18.22015 exknots = -13.2689 28.58215 degree = 3);
output out = xx(drop = x_0);
id lag;
run;
/*The macro below uses the same arguments as the score macro plus reference temperature
and names of the two output data sets used to produce graphs illustrating local (Des1) and total
(Des2) effects of temperature.
Note that the reference temperature must be represented in the input temperature data*/
%macro cross_score(inx=,inlag=,inxcat=,inlagcat=,xsyntax=,lagsyntax=,ref=,Des1=,Des2=);
proc sql noprint;
select distinct name into :listx separated by ' ' from dictionary.columns where
libname = "%upcase(&inxcat)"  and memname = "%upcase(&inx)" and
upcase(name) contains "%upcase(&xsyntax)" order by varnum;
select distinct name into :listlag separated by ' ' from dictionary.columns where
libname = "%upcase(&inlagcat)" and memname = "%upcase(&inlag)" and
upcase(name) contains "%upcase(&lagsyntax)" order by varnum;
create table &Des1 as select distinct
%let i = 1;
%do %while (%scan(&listx,&i,' ') ne %str());
%let j = 1;
%do %while (%scan(&listlag,&j,' ') ne %str());
(a.%scan(&listx,&i,' ')-c.%scan(&listx,&i,' '))*b.%scan(&listlag,&j,' ') as
%scan(&listx,&i,' ')_%scan(&listlag,&j,' ') ,
%let j = %eval(&j+1);
%end;
%let i = %eval(&i+1);
%end;
```

```
a.x,a.lag
from
&inx as a inner join &inlag as b on a.lag = b.lag
inner join &inx as c on a.lag=c.lag
where c.x = &ref
order by a.x,a.lag;
create table &Des2 as select distinct
%let i = 1;
%do %while (%scan(&listx,&i,' ') ne %str());
%let j = 1;
%do %while (%scan(&listlag,&j,' ') ne %str());
sum((a.%scan(&listx,&i,' ')-c.%scan(&listx,&i,' '))*b.%scan(&listlag,&j,' ')) as
%scan(&listx,&i,' ')_%scan(&listlag,&j,' ') ,
%let j = %eval(&j+1);
%end;
%let i = %eval(&i+1);
%end;
a.x
from
&inx as a inner join &inlag as b on a.lag = b.lag
inner join &inx as c on a.lag=c.lag
where c.x = &ref
group by a.x
order by a.x;
quit;
%mend;
/*Run cross_score macro with reference temperature at 14.3*/
%cross_score(inx=xx,inlag=laglag,inxcat=work,inlagcat=work,xsyntax=x_,lagsyntax=lag_,ref=
14.3,Des1=Local,Des2=Total);
/*SAS offers many ways of combining these macros and the data they produce into graphs
as those presented in the paper. One easy way is to merge (on date) what comes
out of the cross macro with outcome data and data containing co-variables,
regress outcome on variables from the cross macro and co-variables and
record estmates of relevant regression parameters and the corresponding covariance estimate.
Import regression parameters and covariance estimate plus what comes
out of the cross_score macro into PROC IML, score the models, export back to SAS and plot output*/
```

Supplemental Tables

Supplemental Table 1: Number of measurements per month in the Malmö Offspring Study cohort (columns 2-4) and average mean temperature and standard deviation of mean temperature for lag-periods of 21 days in Malmö ending in the respective month (columns 5-6).

Month	Plasma copeptin	Urine osmolality	Water intake	Average	Standard deviation
January	346	349	275	-0.64	2.61
February	423	455	343	-1.08	2.37
March	431	460	318	0.17	2.14
April	273	320	253	3.61	2.49
May	296	334	260	8.94	2.90
June	353	329	248	13.74	2.73
July	154	179	120	17.25	2.63
August	346	341	243	18.73	2.47
September	473	517	404	15.25	2.54
October	599	585	466	9.94	2.82
November	650	636	514	5.17	2.87
December	379	412	325	1.01	2.60

Supplemental Figures

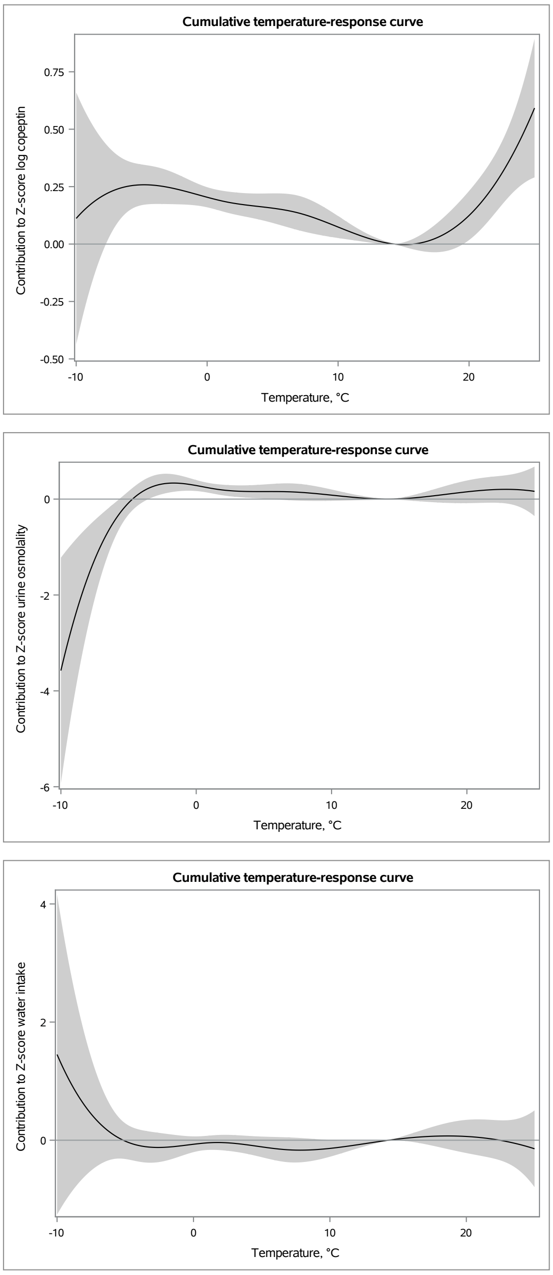

Supplemental Figure 1: Cumulative effect of temperature on different indicators of fluid homeostasis (plasma copeptin (**all cohorts**), urine osmolality (**Malmö Offspring Study cohort only**) and water intake (**Malmö Offspring Study cohort only**)) during entire period of 21 days. All presented relative to reference temperature at 14.3 °C. The grey zones denote the 95% confidence intervals. Please note that the y-axis varies across panels.

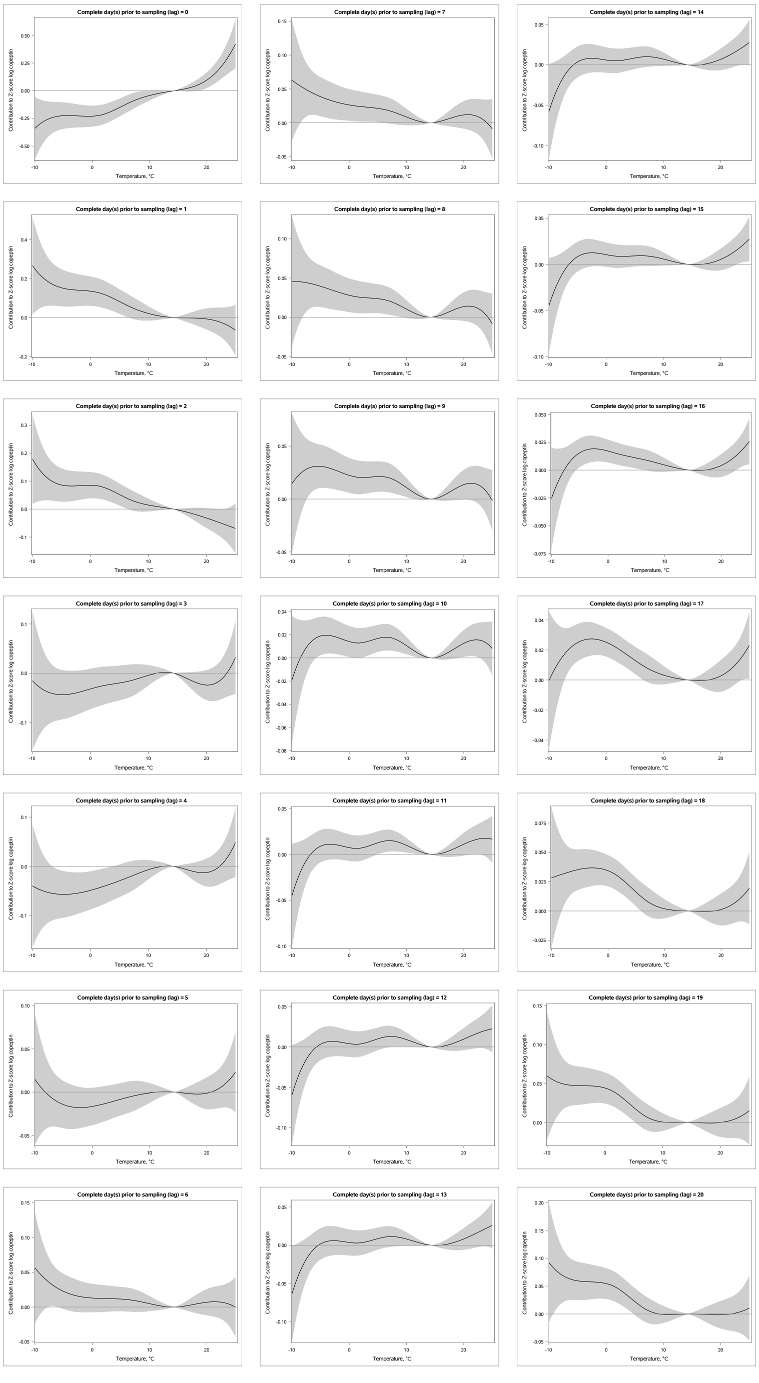

Supplemental Figure 2: Effect of past outdoor temperature on plasma copeptin (**all cohorts**). Effect of temperature at different number of complete days prior sampling. All presented relative to reference temperature at 14.3 °C. The grey zones denote the 95% confidence intervals. Please note that the y-axis varies across panels.

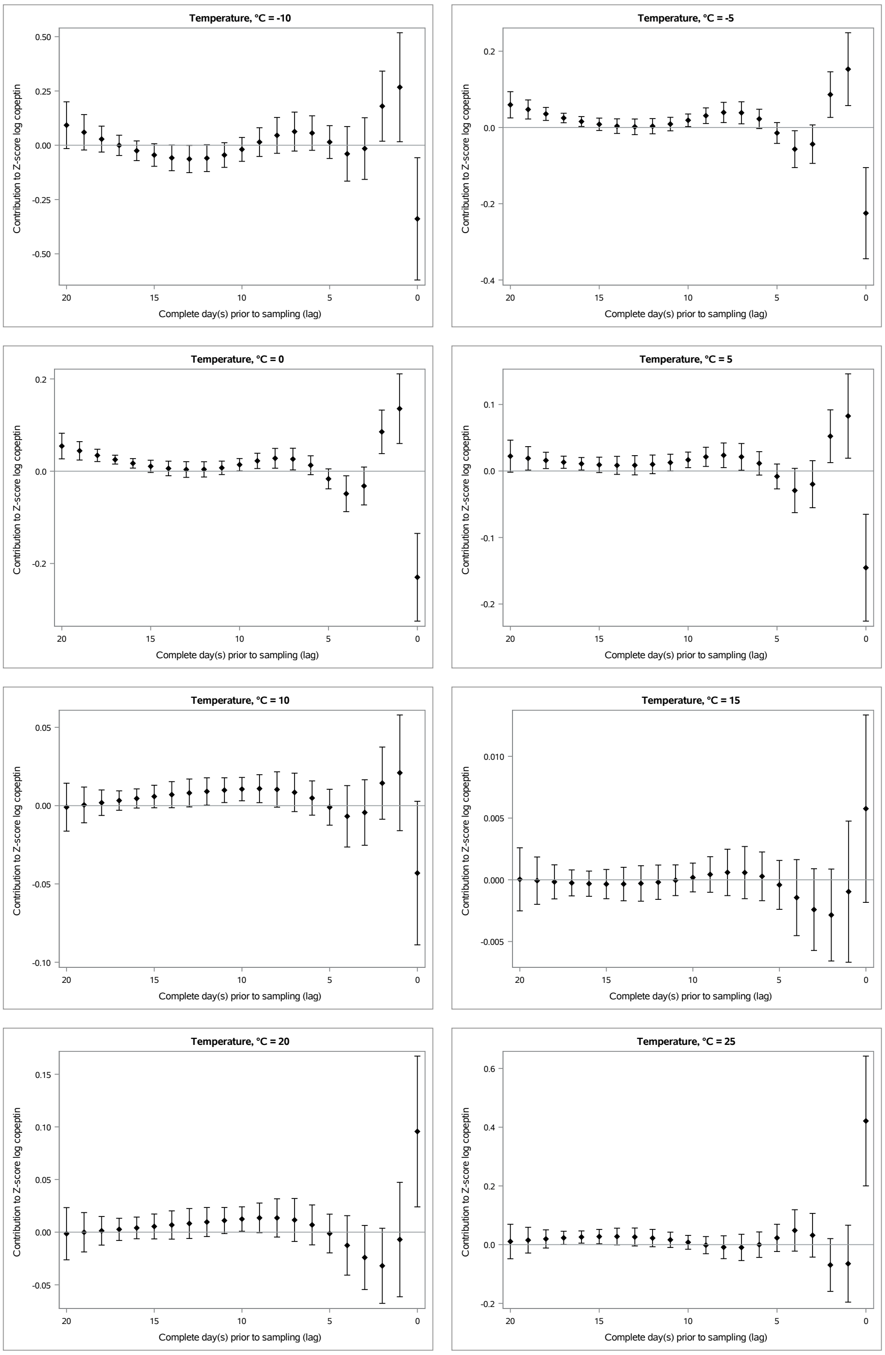

Supplemental Figure 3: Effect of past outdoor temperature on plasma copeptin (**all cohorts**). Effect of temperature at different number of complete days prior sampling. All presented relative to reference temperature at 14.3 °C. The bars denote the 95% confidence intervals. Please note that the y-axis varies across panels.

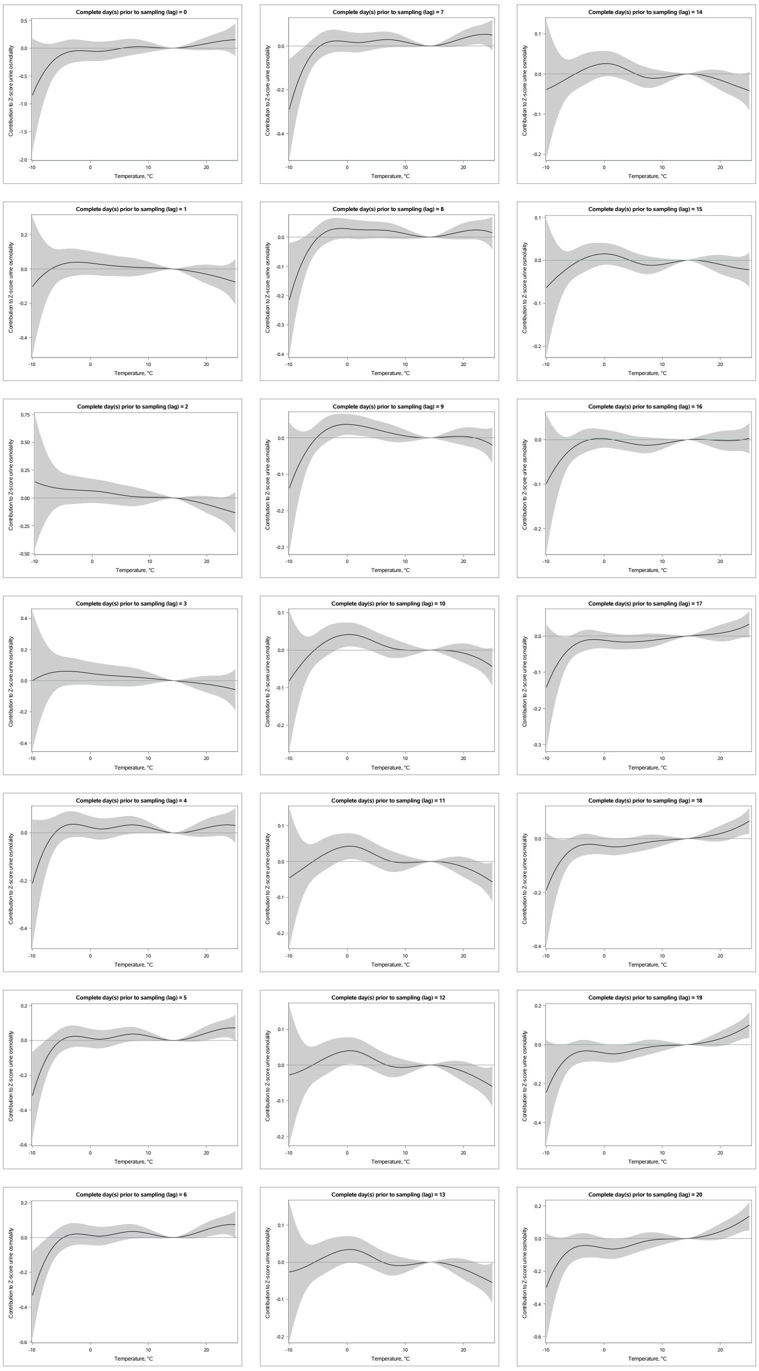

Supplemental Figure 4: Effect of past outdoor temperature on urine osmolality (**Malmö Offspring Study cohort only**). Effect of temperature at different number of complete days prior sampling. All presented relative to reference temperature at 14.3 °C. The grey zones denote the 95% confidence intervals. Please note that the y-axis varies across panels.

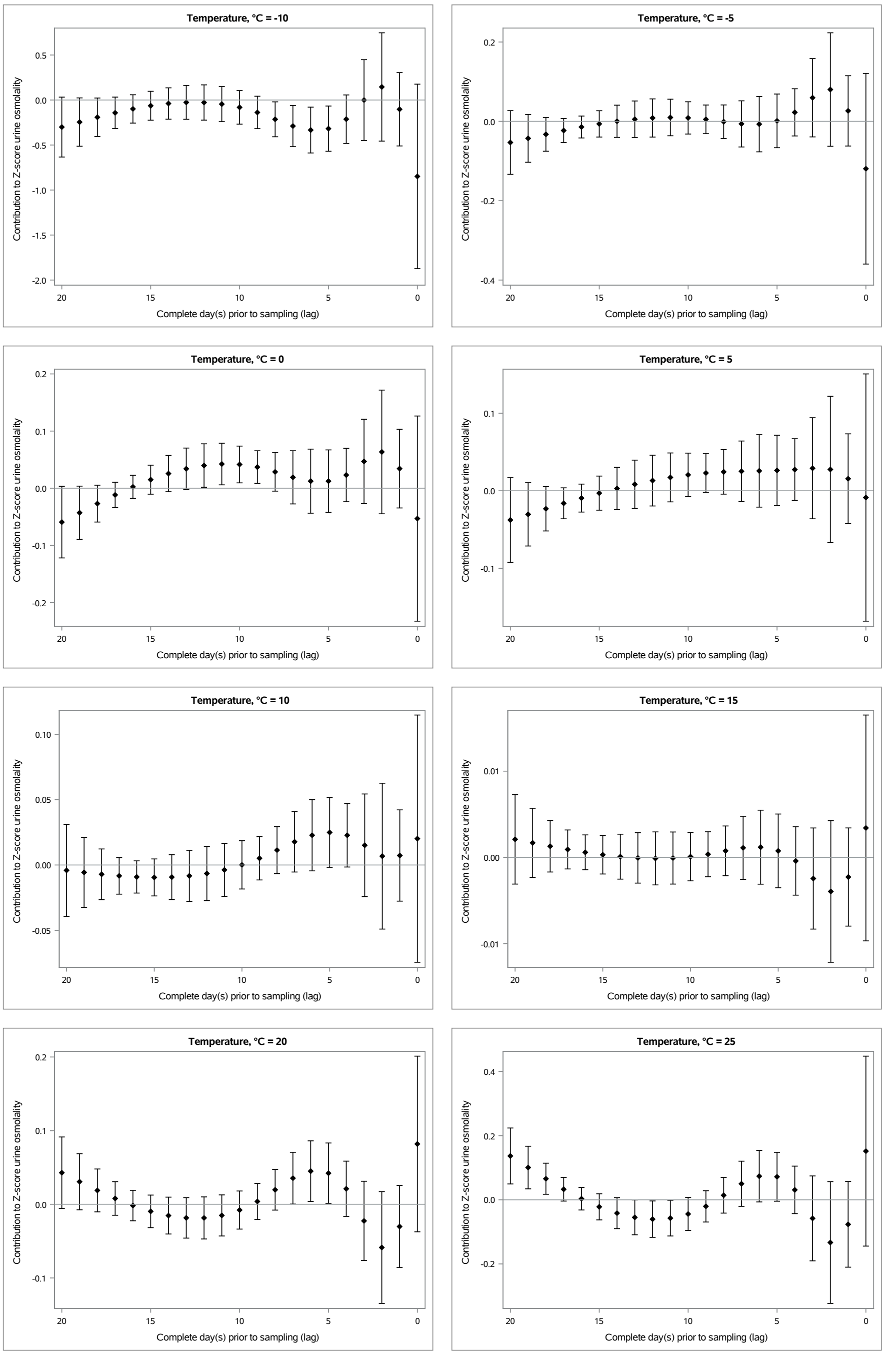

Supplemental Figure 5: Effect of past outdoor temperature on urine osmolality (**Malmö Offspring Study cohort only**). Effect of temperature at different number of complete days prior sampling. All presented relative to reference temperature at 14.3 °C. The bars denote the 95% confidence intervals. Please note that the y-axis varies across panels.

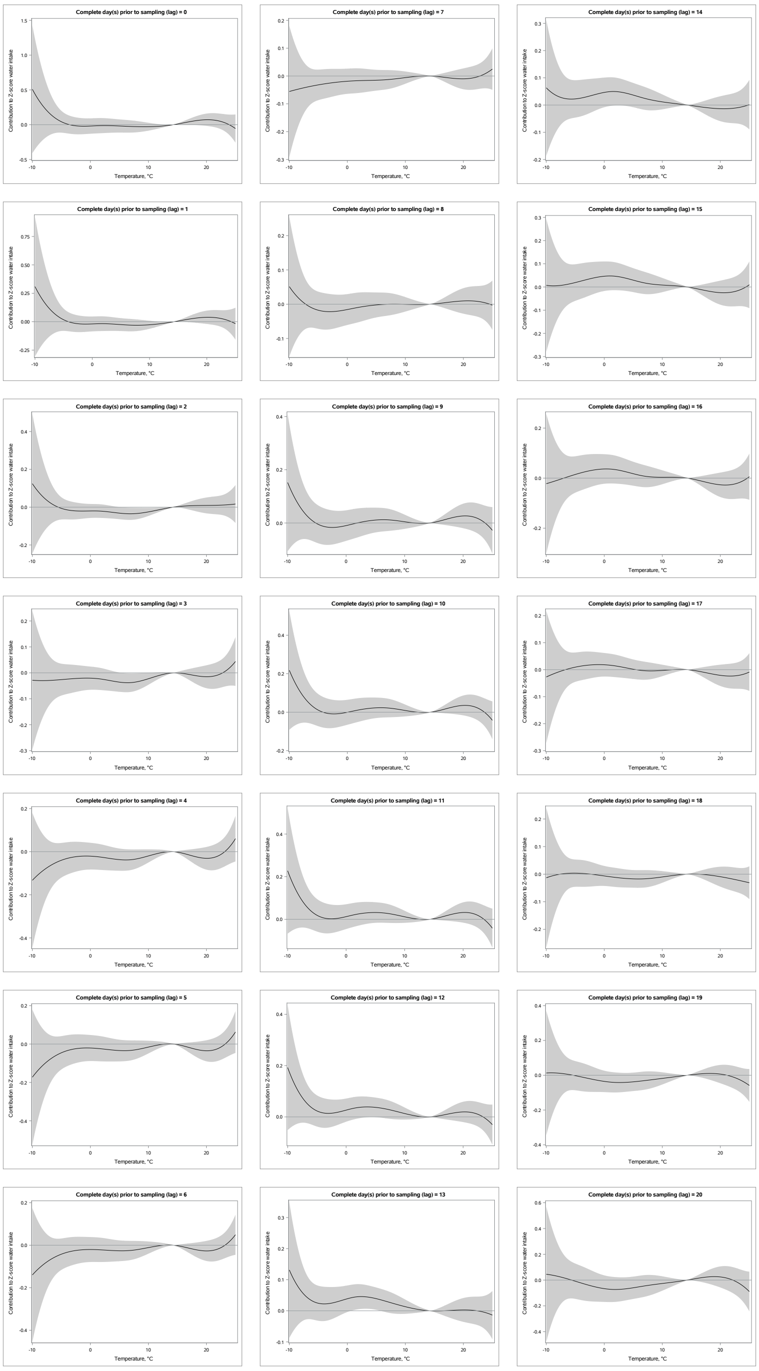

Supplemental Figure 6: Effect of past outdoor temperature on water intake (**Malmö Offspring Study cohort only**). Effect of temperature at different number of complete days prior sampling. All presented relative to reference temperature at 14.3 °C. The grey zones denote the 95% confidence intervals. Please note that the y-axis varies across panels.

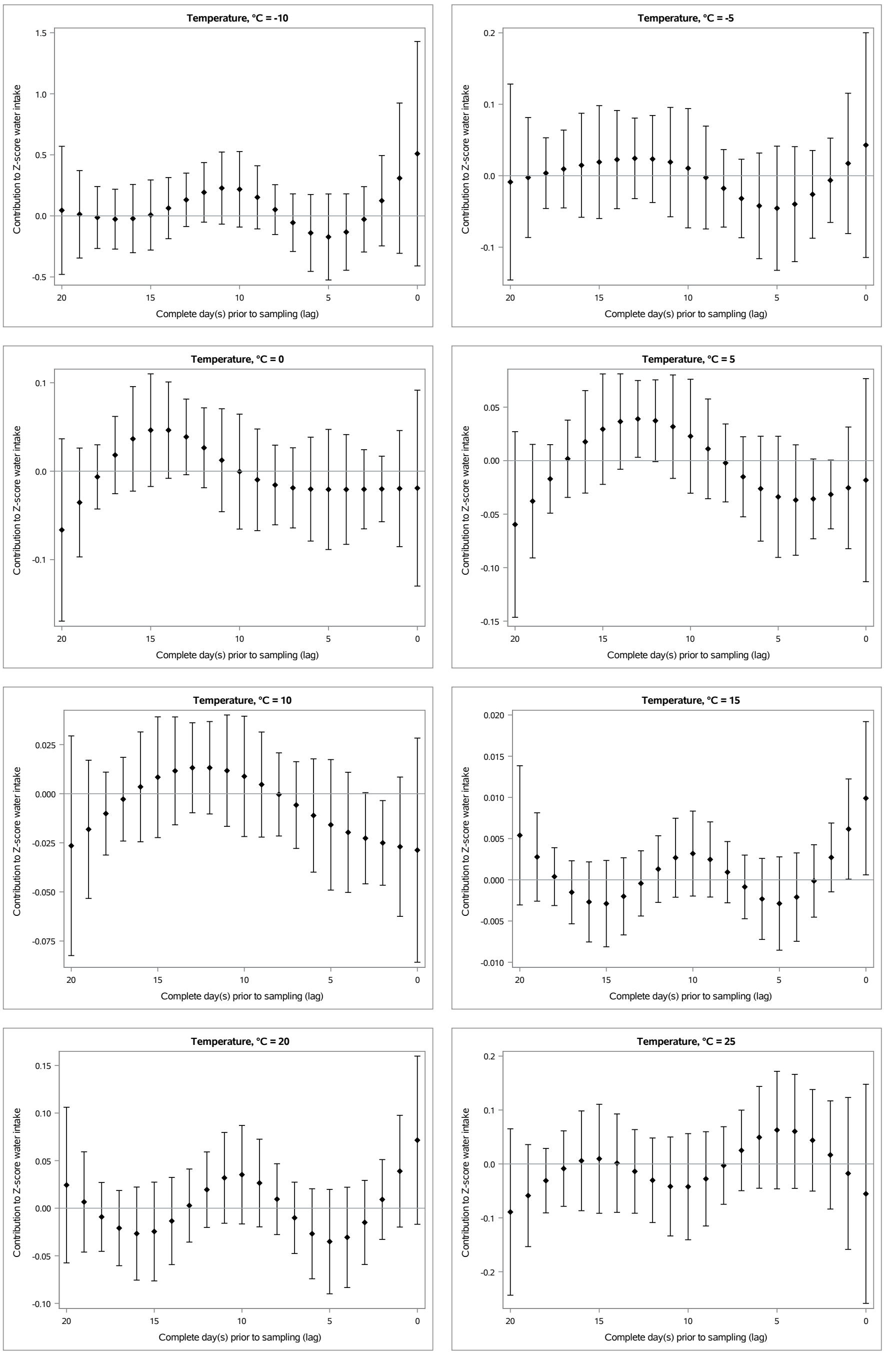

Supplemental Figure 7: Effect of past outdoor temperature on water intake (**Malmö Offspring Study cohort only**). Effect of temperature at different number of complete days prior sampling. All presented relative to reference temperature at 14.3 °C. The bars denote the 95% confidence intervals. Please note that the y-axis varies across panels.

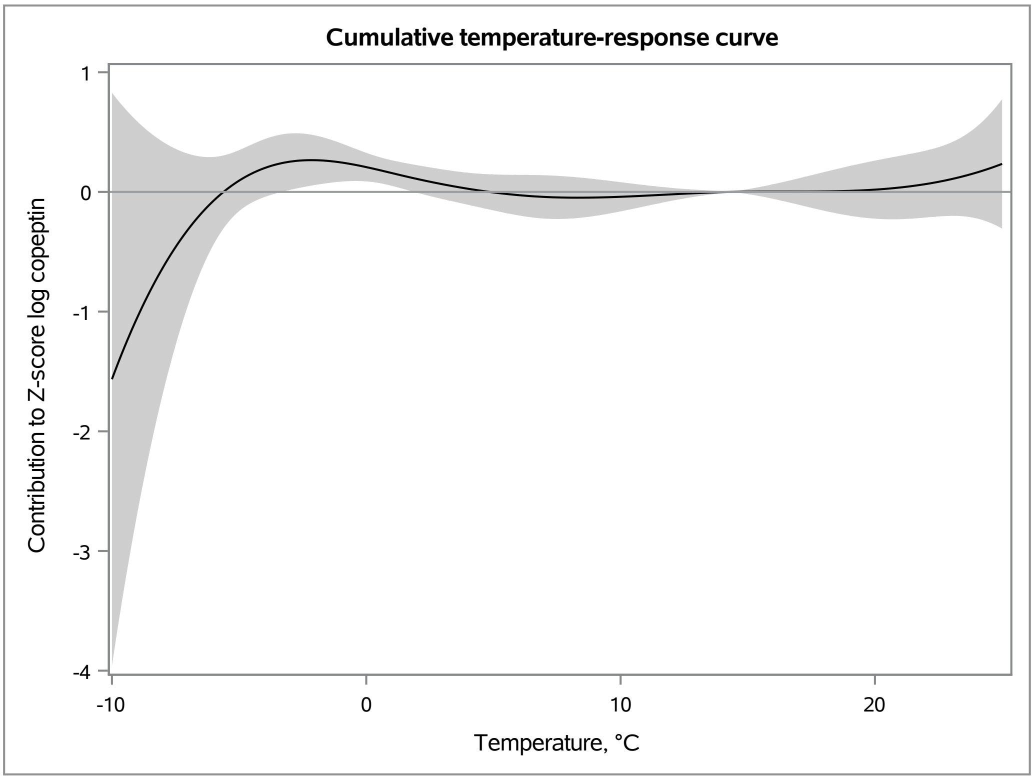

Supplemental Figure 8: Cumulative effect of temperature on plasma copeptin during entire period of 21 days in the Malmö Offspring Study cohort. Separate analysis in the Malmö Offspring Study cohort only. All presented relative to reference temperature at 14.3 °C. The grey zone denotes the 95% confidence intervals.

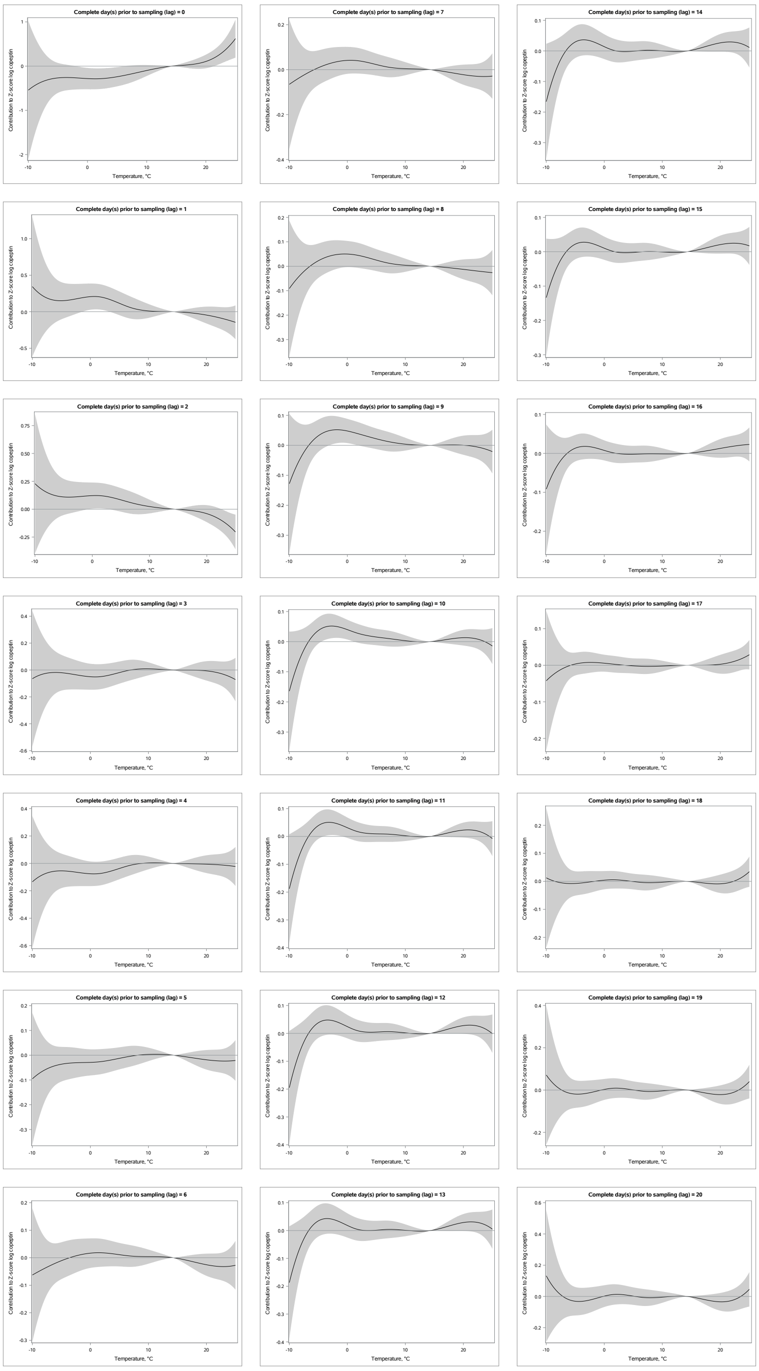

Supplemental Figure 9: Effect of past outdoor temperature on plasma copeptin in the Malmö Offspring Study cohort. Separate analysis in the Malmö Offspring Study cohort only. Effect of temperature at different number of complete days prior sampling. All presented relative to reference temperature at 14.3 °C. The grey zones denote the 95% confidence intervals. Please note that the y-axis varies across panels.

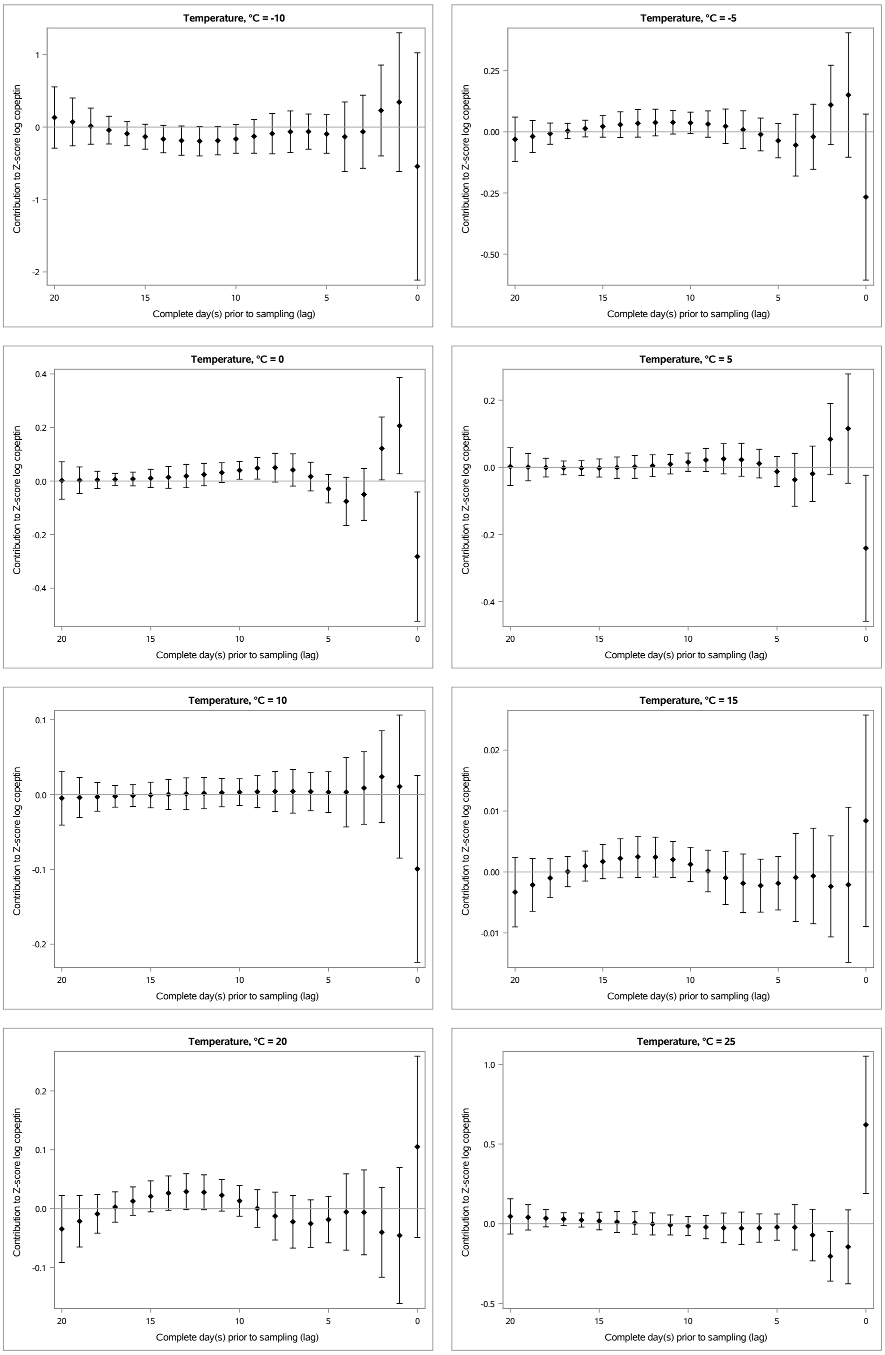

Supplemental Figure 10: Effect of past outdoor temperature on plasma copeptin in the Malmö Offspring cohort. Separate analysis in the Malmö Offspring cohort only. Effect of temperature at different number of complete days prior sampling. All presented relative to reference temperature at 14.3 °C. The bars denote the 95% confidence intervals. Please note that the y-axis varies across panels.

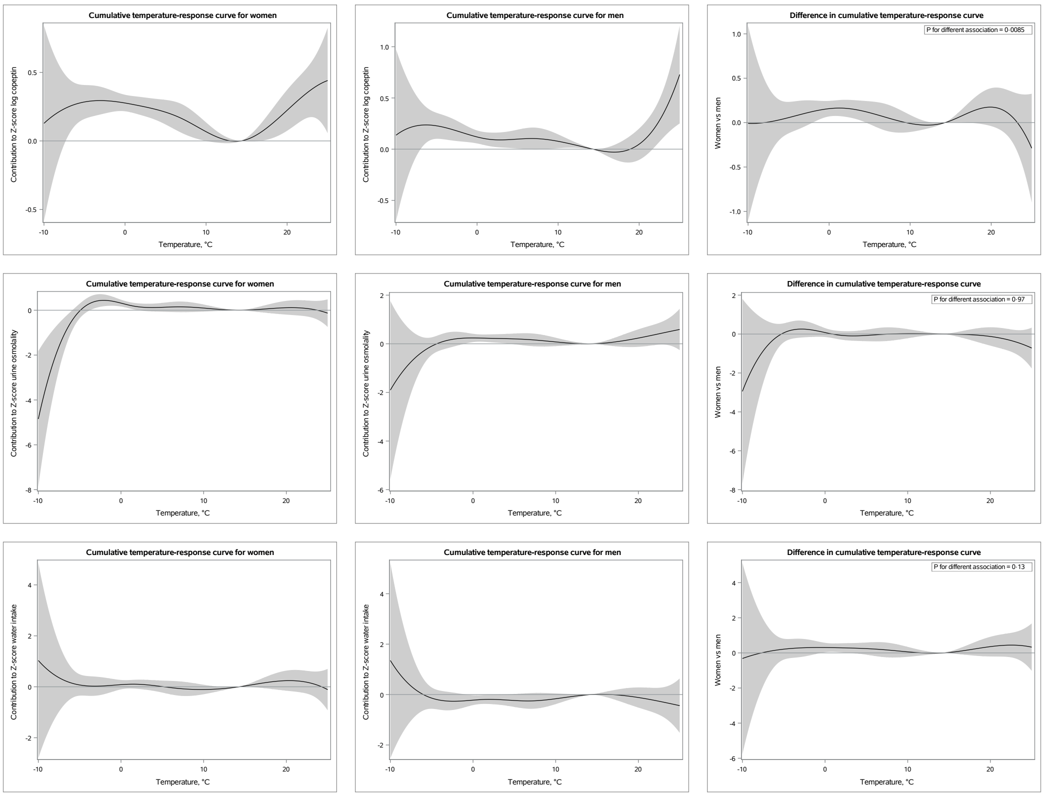

Supplemental Figure 11: Cumulative effect of temperature on different indicators of fluid homeostasis (plasma copeptin (**all cohorts**), urine osmolality (**Malmö Offspring Study cohort only**) and water intake (**Malmö Offspring Study cohort only**)) during entire period of 21 days in women (far left panels) and men (middle panels), respectively, as well as difference between the effects of women and men (far right panels). The grey zones denote the 95% confidence intervals. Please note that the y-axis varies across panels.

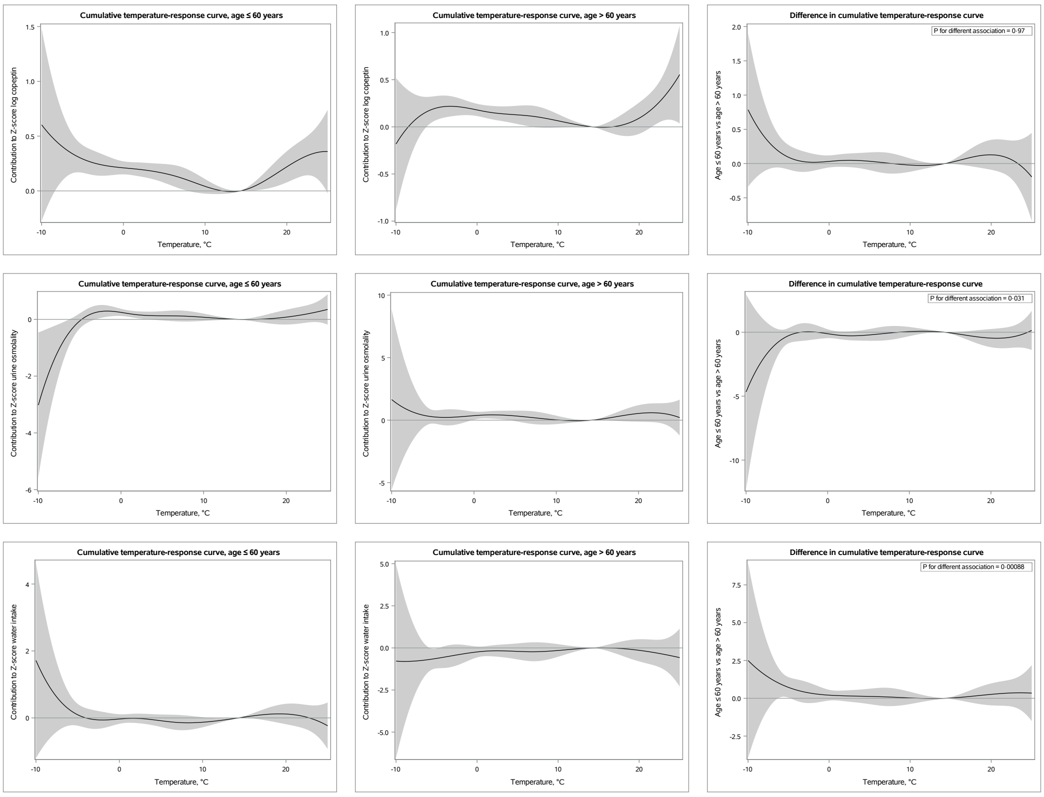

Supplemental Figure 12: Cumulative effect of temperature on different indicators of fluid homeostasis (plasma copeptin (**all cohorts**), urine osmolality (**Malmö Offspring Study cohort only**) and water intake (**Malmö Offspring Study cohort only**)) during entire period of 21 days in individuals aged ≤ 60 years (far left panels) and > 60 years (middle panels), respectively, as well as difference between the effects of the two age groups (far right panels). The grey zones denote the 95% confidence intervals. Please note that the y-axis varies across panels.

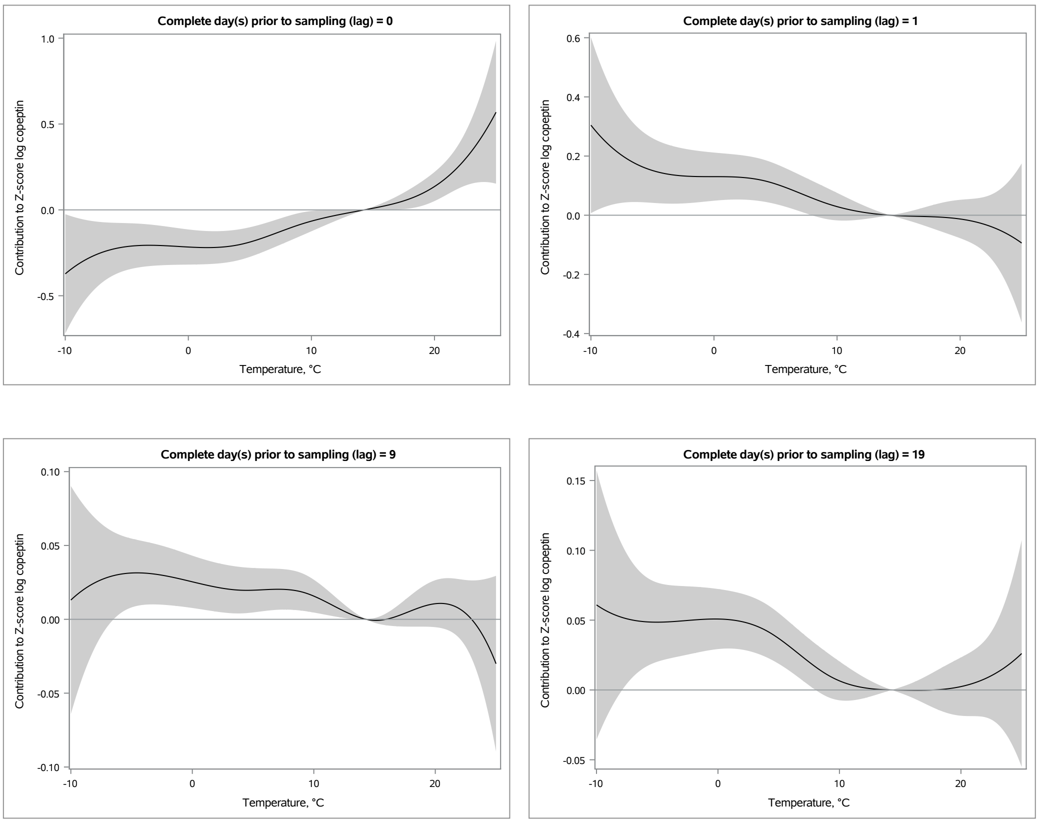

Supplemental Figure 13: Effect of absolute temperature on plasma copeptin (**all cohorts**) at different number of complete days prior blood sampling. All presented relative to reference temperature at 14.3 °C. The grey zones denote the 95% confidence intervals. Please note that the y-axis varies across panels.

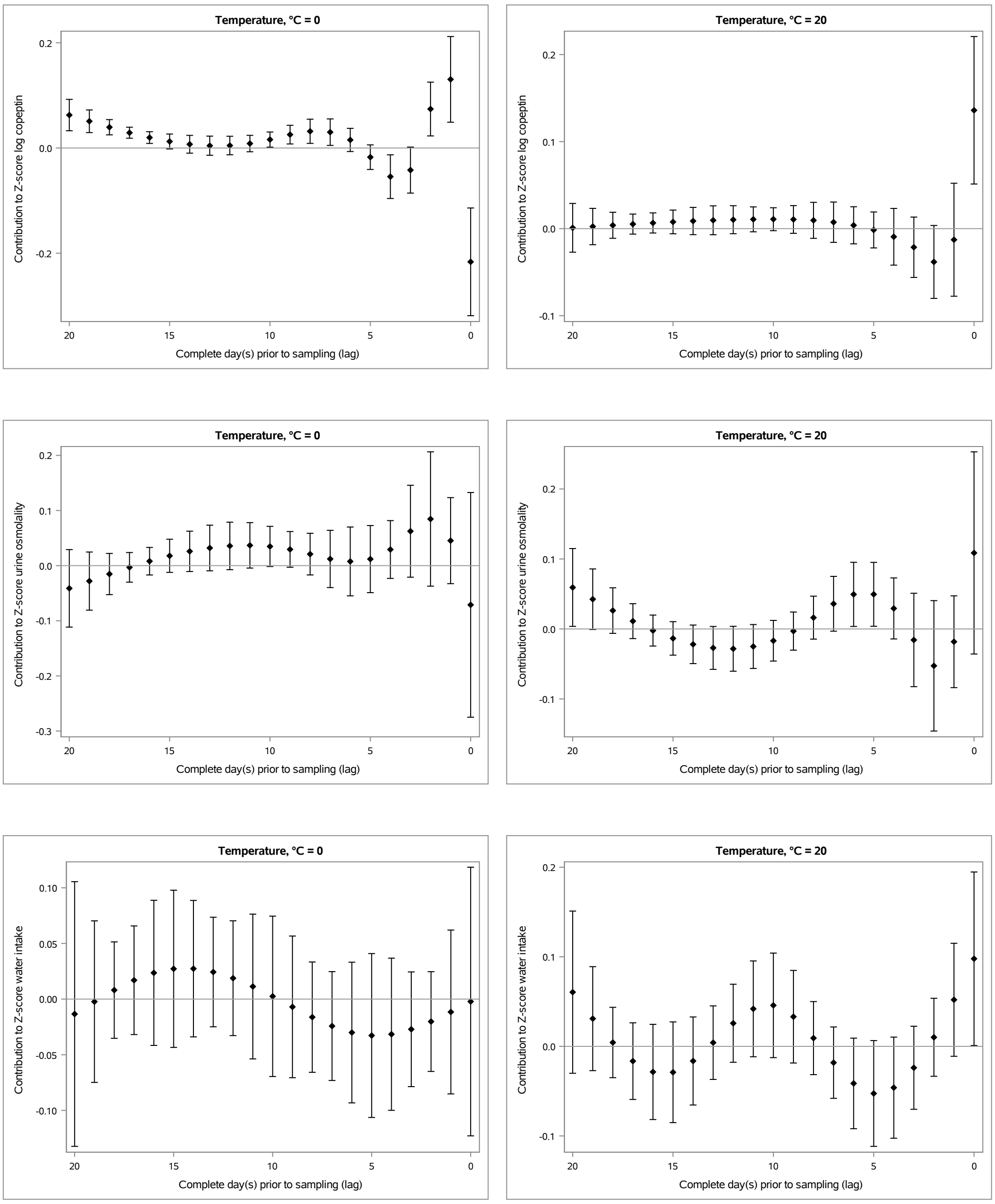

Supplemental Figure 14: Effect of absolute temperatures of 0 °C and 20 °C on indicators of fluid homeostasis. Effect of absolute temperatures of 0 °C and 20 °C on outcome (plasma copeptin (**all cohorts**), urine osmolality (**Malmö Offspring Study cohort only**) and water intake (**Malmö Offspring Study cohort only**)) at different number of complete days prior sampling. All presented relative to reference temperature at 14.3 °C. The bars denote the 95% confidence intervals. Please note that the y-axis varies across panels.

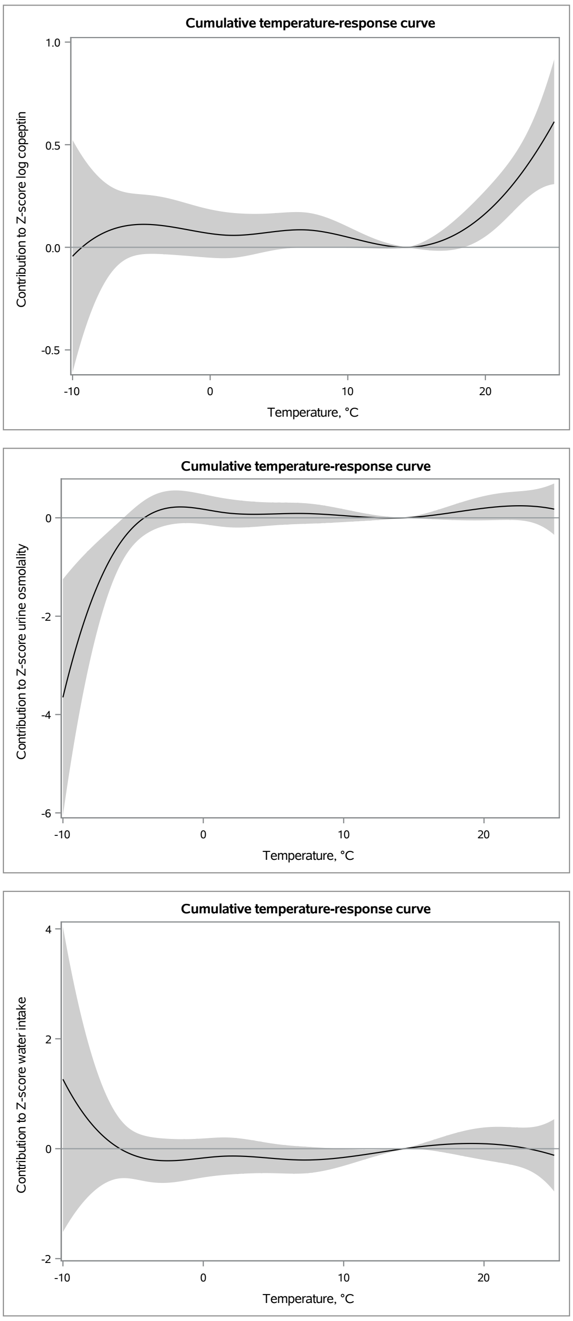

Supplemental Figure 15: Cumulative effect of temperature with adjustment for seasonality on different indicators of fluid homeostasis (plasma copeptin (**all cohorts**), urine osmolality (**Malmö Offspring Study cohort only**) and water intake (**Malmö Offspring Study cohort only**)) during entire period of 21 days. All presented relative to reference temperature at 14.3 °C. The grey zones denote the 95% confidence intervals. Please note that the y-axis varies across panels.

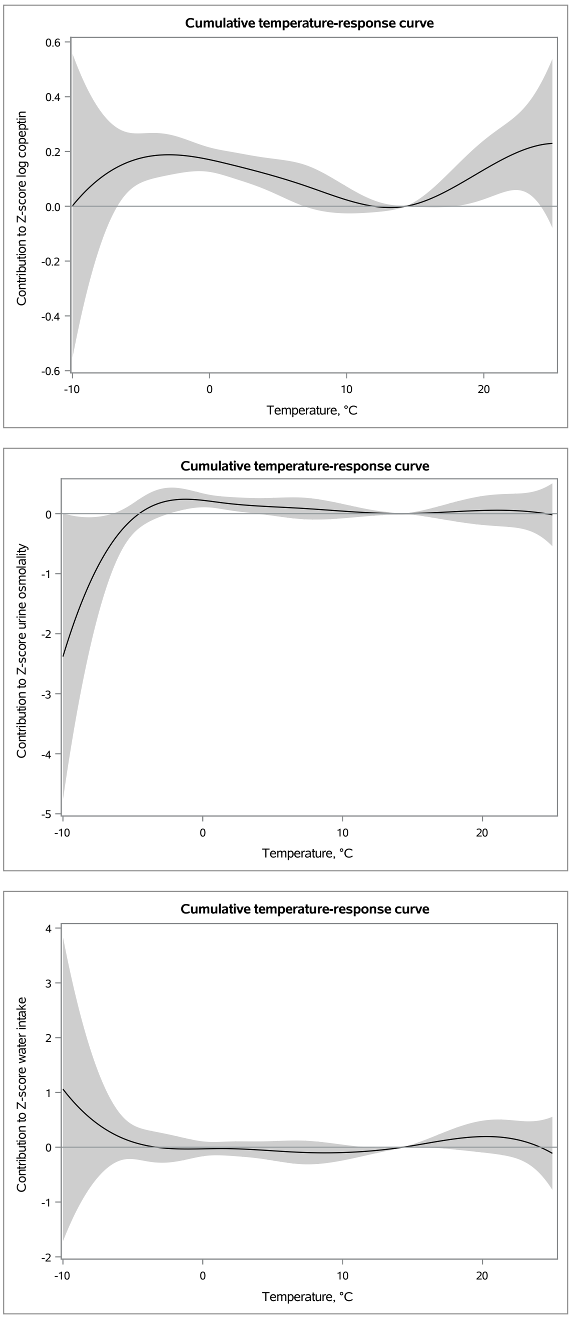

Supplemental Figure 16: Cumulative effect of temperature with adjustment for time trend on different indicators of fluid homeostasis (plasma copeptin (**all cohorts**), urine osmolality (**Malmö Offspring Study cohort only**) and water intake (**Malmö Offspring Study cohort only**)) during entire period of 21 days. All presented relative to reference temperature at 14.3 °C. The grey zones denote the 95% confidence intervals. Please note that the y-axis varies across panels.

Supplemental References

[1] Katsouyanni K. *et al.* Short term effects of air pollution on health: a European approach using epidemiologic time series data: the APHEA protocol. Journal of Epidemiology and Community Health 1996; 50(Suppl 1):S12-S18

[2] Gasparrini A. *et al.* Distributed lag non-linear models. Statistics in Medicine. 2010; 29:2224-34.
